# Regional coronary endothelial function is related to local coronary wall thickness in CAD patients using 3T MRI

**DOI:** 10.1186/1532-429X-13-S1-P242

**Published:** 2011-02-02

**Authors:** Allison G Hays, Sebastian Kelle, Glenn A Hirsch, Jing Yu, Harsh K Agarwal, Gary Gerstenblith, Matthias Stuber, Robert G Weiss

**Affiliations:** 1 Johns Hopkins, Baltimore, MD, USA; 2 Deutsches Herzzentrum Berlin, Berlin, Germany; 3 University of Lausanne, Lausanne, Switzerland

## Background

Coronary endothelial function (endoFx) is an important physiologic indicator of vascular function. Abnormal function is present in patients with established coronary disease (CAD) and was recently shown to relate to the severity of luminal stenosis.^1^ However, luminal stenosis occurs late in the process of atherosclerosis, and a the extent to which abnormal coronary endoFx occurs early in atherosclerosis and whether it is related to measures of early, non-stenotic atherosclerosis, such as coronary wall thickness (CWT) are not known. Recent advances in MRI now allow non-invasive assessment of both anatomic (CWT) ^2-4^ and functional (endoFx) vascular properties that in the past required invasive studies, precluding evaluation of healthy subjects and low risk populations.

## Objective

To test the hypothesis that local endothelial dependent coronary vasoreactivity is inversely related to coronary wall thickness as measured using MRI.

## Methods

Fourteen arteries in eleven healthy adults and fourteen arteries in eleven CAD patients with <30% stenosis by recent coronary x-ray angiography were studied with 3T MRI. To measure endoFx, spiral coronary MRI was performed before and during isometric hand-grip exercise, an endothelial-dependent stressor, and coronary cross-sectional area (CSA) change was measured as previously reported.^1^ For coronary vessel wall imaging, black-blood dual-inversion spiral imaging was used to quantify CWT and normalized wall index (NWI=lumen area/total area).

## Results

**Figure 1:**
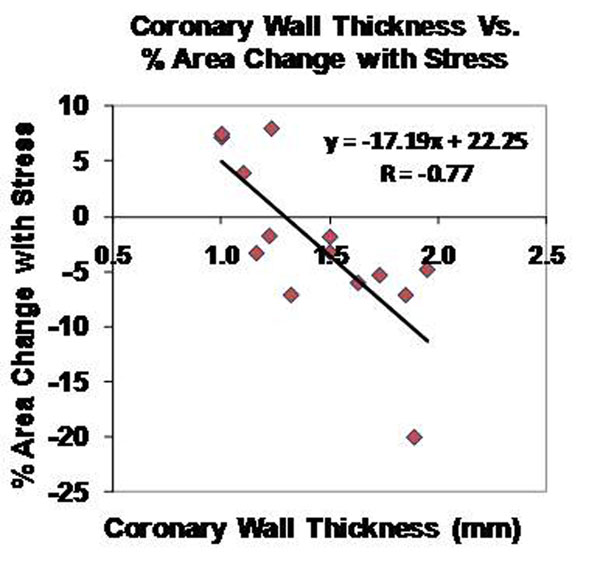
MRI measures of coronary wall thickness (mm) vs. % coronary area change with isometric handgrip stress in CAD patients.

The stress-induced change in CSA was significantly higher in healthy adults (15.2%±11.0%, mean±SD) than in those with mild, non-stenotic CAD (-2.3%±7.4%, p<0.0001). Mean CWT was smaller in healthy subjects (0.86±0.16mm) than in CAD patients (1.49±0.31mm, p<0.0001). In contrast to healthy subjects, coronary endoFx correlated inversely with wall thickness in mild CAD patients (%CSA vs CWT, r=-0.77, p<0.001 (Fig 1) and %CSA vs. NWI, r=-0.70, p=0.005).

## Conclusion

Both anatomic (CWT) and functional (endoFx) coronary changes can be detected using MRI before the development of stenotic disease and the functional abnormalities are related to the extent of local, early anatomic atherosclerosis (CWT). These findings demonstrate that even at the earliest stage of anatomic coronary atherosclerosis, endothelial-dependent functional changes are present and therefore, could contribute to the local progression of atherosclerosis.
